# Foreign body reaction simulating mandibular osteosarcoma—Case report

**DOI:** 10.1016/j.ijscr.2019.03.058

**Published:** 2019-06-08

**Authors:** Gabriela Caovilla Felin, João Paulo De Carli, Mateus Ericson Flores, Jose Luiz Bernardon Pretto, Letícia Copatti Dogenski, Ferdinando De Conto

**Affiliations:** a Department of Oral Surgery, Faculty of Dentistry, University of Passo Fundo, Passo Fundo, RS, Brazil; b Departments of Oral Medicine and Prosthodontics, Faculty of Dentistry, University of Passo Fundo, Passo Fundo, RS, Brazil; c Department of Radiology, Faculty of Dentistry, University of Passo Fundo, Passo Fundo, RS, Brazil; d Faculty of Dentistry of University of Passo Fundo, Passo Fundo, RS, Brazil

**Keywords:** Granuloma, Foreign-body, Alloplastic materials, Osteosarcoma, Case report

## Abstract

**Introduction:**

This study aimed to report the case of a foreign body granuloma in a 34-year-old woman, presenting clinical and imaging characteristics, as well as to discuss the association of using alloplastic material for the lesion in question.

**Presentation of case:**

Female patient seeking dental care for a routine treatment. The panoramic radiograph showed the presence of asymptomatic, radiolucent, and multiloculated lesion between teeth 31 and 34. Among the hypotheses diagnosed, central giant cell granuloma and osteosarcoma were suggested. When questioned, the patient denied any health changes or recent local procedures. After performing a histopathological examination and insisting on anamnesis, the case was diagnosed more accurately as "foreign body reaction" and the adequate therapy was determined.

**Discussion:**

The use of alloplastic material has been reported as an etiological factor of foreign body lesions, and dentists, either clinicians, radiologists or pathologists, should be aware of this because of the great increase of such procedures and the difficulties related to their diagnosis.

**Conclusion:**

The foreign body granuloma associated with silicone prosthesis, based on its clinical and imaging characteristics, may be mistaken for neoplastic processes.

## Highlights

• Foreign body granulomas may be associated with silicone prostheses.
• Foreign body granulomas can be mistaken for malignant neoplasms.
• Diagnosis of foreign body granuloma needs clinical, imaging and histological analysis.

## 1 Introduction

Currently, attempting to prevent aesthetic changes caused by aging, the use of injectable soft tissue fillers is daily observed. Initially used for the treatment of expression lines, the concept of fills has expanded to include the correction of the loss of volume of the aged face [1]. Allogenic implants are an alternative for reconstructing facial defects, and they are used to restore a variety of tissue defects [2]. However, these materials are easy to use and cause few side effects [3].

Although dermal fillers are generally considered safe, they are not free of risks or complications. The true incidence of complications is difficult to establish, given the lack of a universal reporting system, and it is likely that many minor complications are simply not brought to the attention of health professionals [4]. Several unwanted effects may occur from prosthesis insertion, such as edema, migration, allergic response, and nerve paralysis [5]. Foreign body granulomas may also develop after injecting cosmetic fillers in the facial area [6].

Granulomas are microscopic structures composed of macrophages, surrounded by lymphocytes and filled with collagen fibers and fibroblasts [7]. Although they can occur with all injectable dermal fillers, the incidence is very rare (0.01–1.0%) and usually appears after a latency period, which may be several months to years after the injection [8].

Different from foreign body granuloma, the mandibular osteosarcoma is a rare malignant condition that represents approximately 6%–7% of osteosarcomas and 1% of all malignant head and neck neoplasias [9]. The exact cause is unknown, but there are several predisposing factors that may cause osteosarcoma. In the oral cavity this tumor is slightly more common in men [10] and usually affects patients 10–20 years older than those affected by osteosarcomas of long bones, presenting a lower incidence of distant metastasis [11].

This study aimed to report the case of a foreign body granuloma in a 34-year-old woman, presenting clinical and imaging characteristics, as well as to discuss the association of using alloplastic material for the lesion in question. This work has been reported in line with the SCARE criteria [12].

## 2 Presentation of case

**Fig. 1:**
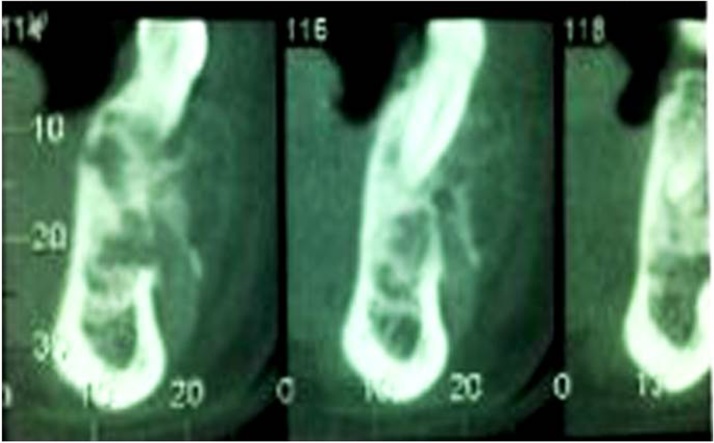
Cone beam computed tomography with image of the lesion and hypodense image in the chin region.

Female patient, 34 years old, caucasian, whose routine tomographic examination detected multiloculated hypodense lesion in the region of elements 31–34 (Fig. 1).

In the intraoral physical examination, the patient presented buccal bulging, without complaining of pain or loss of vitality in the dental elements. When questioned, the patient reported a mild local trauma occurred 30 days earlier. Laboratory tests for calcium, alkaline phosphatase, and parathyroid hormone were requested and did not show alterations.

**Fig. 2:**
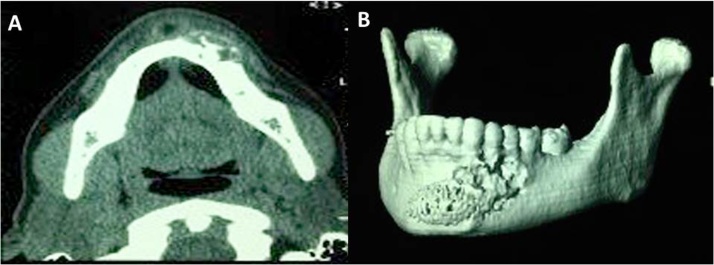
A – Axial cut of the computed tomography showing medullary and cortical bone destruction, not affecting the inner mandibular cortical surface. B – Computed tomography with 3D reconstruction, showing the alloplastic material in the chin region. Because the patient concealed such information, the image identified suggested a bone tumor lesion, either malignant or with aggressive local behavior, of odontogenic or non-odontogenic mandibular origin.

The imaging examinations revealed, in the panoramic radiograph, the presence of circumferential radiolucent area with precise limits between dental elements 33 and 34, in the middle third and apical regions. Computed tomography showed an extensive hypodense image of 2.0 cm, unclear, expansive, and with undetermined aspect, located in the region of dental elements 32, 33, and 34. It also infiltrated the anterior myoadipose surfaces in the chin and oral space regions, suggesting a bone tumor lesion either malignant or with local aggressive behavior (e.g., osteosarcoma) (Fig. 2A and B).

**Fig. 3:**
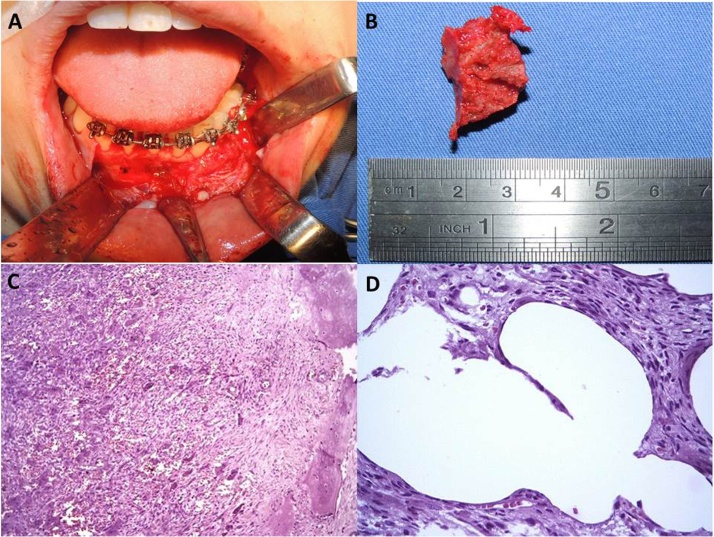
A – Incisional biopsy procedure. B – Fragment of tissue removed during the incisional biopsy procedure. C – Microscopy of the previous biopsy. HE: dimension 100x. The field shows several fragments of white and contoured tissue with irregular and friable consistency, as well as giant multinucleated cells, macrophages, and bone neoformation, suggesting giant tumor cells. D – HE: 400×. Presence of clear vacuoles compatible with fragments of previous silicone with giant multinucleated cells in the edges.

Considering this condition, an incisional biopsy of the lesion was performed under local anesthesia, in surgical block, which result showed a central giant cell granuloma (Fig. 3A–D). The team was composed of two PhD and a specialist in Buccomaxillofacial Surgery.

**Fig. 4:**
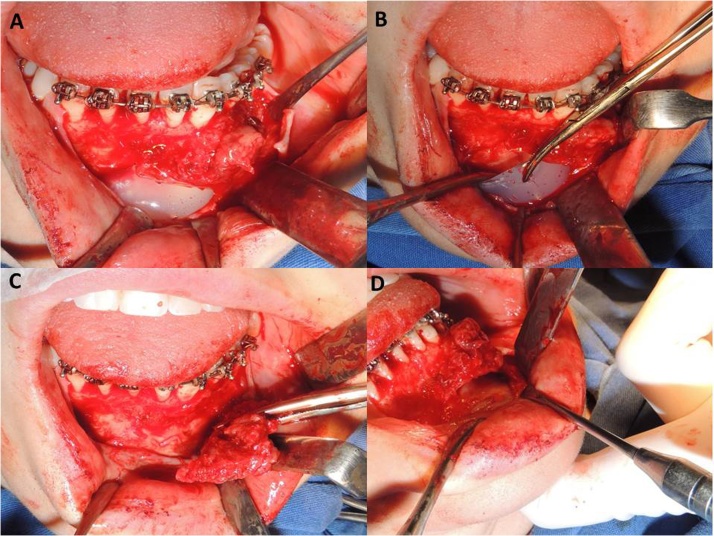
A and B – During the surgical procedure, the silicone prosthesis was identified and removed. C – Curettage and removal of the granulation tissue. D – The extension of the lesion ended up reaching the external bone flap of the mandible.

Based on this result, the surgical approach was applied for lesion curettage, without regional bone resection. Intraoperation revealed the presence of abundant granulation tissue and, overall, identified the presence of foreign body compatible with silicone prosthesis, which excluded clinically the radiographic hypothesis of osteosarcoma or central giant cell granuloma (Fig. 4A–D). Thus, it was decided for the curettage of the entire lesion, also removing the prosthesis.

For both biopsies, the patient was instructed to take amoxicillin (500 mg 8/8 h for 7 days) plus ibuprofen (600 mg every 6 h for 5 days). Post-intervention care was performed in the dental office.

**Fig. 5:**
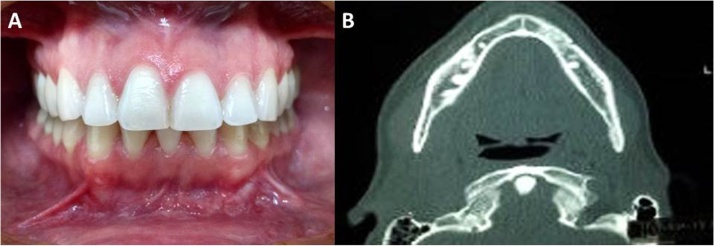
A – After 12 months of clinical control, bone repair and the absence of signs of mandibular lesion were detected at the site. B – Axial cut of the postoperative computed tomography, with discrete change in the trabecular bone in the mandible.

In the postoperative period, when questioned again, the patient reported having been subjected to aesthetic procedure around 10 months earlier, confirming the previous prosthesis installation. After 12 months of clinical and imaging control, bone repair and the absence of any sign of mandibular lesion were confirmed (Fig. 5A and B). The patient was instructed to return annually for postoperative control.

The patient provided a written consent for the publication of this clinical case.

## 3 Discussion

Alloplastic implants are advantageous due to easy removal when using solid silicone, absence of morbidity of the donating site, implant stability, and no resorption [13]. However, these materials present certain risks of complications based on their contour surface, flexibility, and reactivity to the surrounding tissue [2]. Such affirmations agree with the events of the present case report, which showed failure of aesthetic prosthesis inserted in the chin.

Granulomas show preference for female individuals, probably because women seek cosmetic care more often [7]. Tamiolakis et al. [3] reported two cases of foreign body granuloma following the use of soft tissue fillers, presenting a review of the literature about the matter. The authors demonstrated that, of the 104 similar cases published previously, 98.1% affected women. According to these authors [3], the strong predilection for women obviously reflects the fact that they are the ones who most seek cosmetic procedures. Coincidentally, the patient of the present case was a woman.

Foreign body granulomas can occur until 15 years after placing silicone, showing high incidence rate [14]. The time of prosthesis placement of the patient in question was 10 months, which is considered fairly short to cause bone lesion with such a significant involvement.

Imaging characteristics are important diagnostic tools. The visibility of foreign bodies and an osteosarcoma varies according to composition, imaging technique, and the ability of different materials to minimize radiation. Foreign bodies may be visualized depending of their density and proximity to the tissue in which they are inserted. Magnetic resonance imaging is probably the preferred imaging technique to locate foreign bodies embedded in soft tissues [15]. In the present study, magnetic resonance was not the first choice imaging exam because there was initially no suspicion of foreign body insertion at the lesion site. Central giant cell granuloma and osteosarcoma were considered the diagnostic hypotheses, which confirms the importance of planning the treatment of any lesion, combining the history of the patient with the clinical and imaging findings along with the histopathological examination report to offer an improved therapeutic approach.

In cases in which foreign body granulomas are clinically as a nodule, the surgical excision is both diagnostic and therapeutic [3]. In the case hereby studied, considering the lesion had been diagnosed histopathologically as central giant cell granuloma, because the patient had concealed an information during anamnesis, lesion resection was decided as the initial and definitive treatment.

## 4 Conclusion

Foreign body lesions associated with alloplastic materials have been reported due to the increased use of alloplastic materials for filling soft tissues. The foreign body granuloma associated with silicone prosthesis, based on its clinical and imaging characteristics, may be mistaken for neoplastic processes, and the histopathological examination is vitally important, although the transurgical aspect of the lesion also has a great influence on the diagnosis.

## Conflicts of interest

None of the authors has any conflict of interest.

## Sources of funding

The authors state that the present study had no sponsor or source of funding.

## Ethical approval

Because this is a case report, the present study was not appreciated by a research ethics committee. However, it follows as an attached file a patient’s fully informed written consent for publication of the reported clinical case.

## Consent

Written informed consent was obtained from the patient for publication of this case report and accompanying images. A copy of the written consent is available for review by the Editor-in-Chief of this journal on request.

## Author’s contribution

Gabriela Caovilla Felin – Execution of the surgical step; acquisition of data.

João Paulo De Carli – Writing work, discussion and final approval; conception and design of the study.

Mateus Ericson Flores – Analysis and interpretation of imaging exams; analysis and interpretation of data.

Jose Luiz Bernardon Pretto – Execution of the surgical step; acquisition of data.

Letícia Copatti Dogenski – Literature review, translation and spelling revision; conception and design of the study.

Ferdinando De Conto – Execution of the surgical step; acquisition of data.

## Registration of research studies

N/A.

## Guarantor

João Paulo De Carli.

## Provenance and peer review

Not commissioned, externally peer-reviewed.
